# *Phellinus linteus* Grown on Germinated Brown Rice Increases Cetuximab Sensitivity of KRAS-Mutated Colon Cancer

**DOI:** 10.3390/ijms18081746

**Published:** 2017-08-11

**Authors:** Hye-Jin Park, Jeong-Bin Park, Sang-Jae Lee, Minjung Song

**Affiliations:** 1 Department of Food Biotechnology, Gachon University, Kyungji-Do 13120, Korea; nimpi79@hanmail.net; 2 Department of Food Biotechnology, Division of Bioindustry, Silla University, Busan 46958, Korea; parkjb928@daum.net (J.-B.P.); sans76@silla.ac.kr (S.-J.L.); 3 The Research Center for Extremophiles & Marine Microbiology, Silla University, Busan 46958, Korea

**Keywords:** *Phellinuslinteus*, colorectal cancer, Kirsten rat sarcoma viral oncogene homolog, cetuximab, drug sensitivity, β-glucan

## Abstract

Colon cancer is one of the most common types of cancer, and it has recently become a leading cause of death worldwide. Among colon cancers, the v-ki-ras2 Kirsten rat sarcoma viral oncogene homolog (KRAS)-mutated form is notorious for its non-druggable features. Cetuximab, a monoclonal antibody that binds to the epidermal growth factor receptor, has been introduced as an antitumor therapy; however, secondary resistance and side effects significantly limit its effective use in these cancers. In this study, we prepared *Phellinuslinteus* on germinated brown rice (PBR) extracts to increase the sensitivity of KRAS-mutated colon cancers to cetuximab. The combined treatment of PBR extract and cetuximab suppressed SW480 cell viability/proliferation, with the cells exhibiting altered cellular morphology and clonogenic potential. AnnexinV–fluorescein isothiocyanate/propidium iodide–stained flow cytometry and Western blotting were performed, and PBR extract combined with cetuximab treatment increased apoptosis of the SW480 cells and suppressed their KRAS protein expression. The potential of PBR as a synergistic anticancer agent was further investigated in a tumor-xenografted mouse model. Tumor growth was significantly suppressed with PBR extract and cetuximab co-treatment. In conclusion, PBR increased the sensitivity of KRAS-mutated colon cancer cells to cetuximab, which indicates the potential use of PBR as a medical food against colon cancer.

## 1. Introduction

Metastatic colorectal cancer (mCRC) is one of the four major cancer types and is among the leading causes of death in industrialized countries [1]. Colon cancer develops from normal tissue, which transforms to a dysplastic lesion, adenoma, adenocarcinoma, and finally to an invasive adenocarcinoma [2]. During this transformation, the epidermal growth factor receptor (EGFR) pathway plays a major role since it is able to regulate cell growth. EGFR is a member of the Human epidermal growth factor receptor (HER) family of receptor tyrosine kinases (RTKs), and its activation stimulates several signaling cascades, leading to *RAS* gene mutation. Mutant RAS protein activates the mitogen-activated protein kinase (MAPK)/extracellular signal-regulated kinase (ERK) pathway which mediatescell proliferation, metastasis and survival. RAS protein activates other downstream signaling cascades such as the phosphatidylinositol-3-kinase (PI3K)/AKT or c-Jun N terminal kinase (JNK) pathways [3]. Targeting EGFR has been extensively studied in oncology, and monoclonal antibodies (e.g., cetuximab) against the extracellular domain of the EGFR have been developed [4,5] as treatments against cancers, including colorectal cancer [6]. However, this promising therapy was ineffective against v-ki-ras2 Kirsten rat sarcoma viral oncogene homolog (KRAS)-mutated cancers [7,8]. Unfortunately, it is estimated that 30–40% of colon cancer patients have a KRAS mutation, giving them very few therapeutic options [5,9]. Therefore, developing therapies against KRAS-mutated colon cancer is an important, unmet need [10].

In this study, we prepared *Phellinuslinteus* grown on germinated brown rice (PBR) extracts to increase the sensitivity of KRAS-mutated colon cancers to cetuximab. *P. linteus* (Mesima), a fungus of the family Hymenochaetaceae, is a medicinal mushroom, used widely as a traditional Asian medicine to treat stomachache, inflammation, and tumors. Recent studies have shown that theextract of *P. linteus* has anti-inflammatory and antitumor activities [11,12]. Furthermore, proteoglycanpurified from *P. linteus* suppressed colon cancer by protecting T cells and disrupting the EGFR/AKT pathway [13,14], and inhibited SW480 colon cancer cell growth by G2/M phase arrest and suppressed tumor growth in a xenografted model by altering the Wnt/β-catenin pathway [15,16]. However, the availability of *P. linteus* is limited because of supply shortages and high costs. In this study, we grew *P. linteus* on germinated brown rice as an ideal growth medium to address the supply shortage issue [17]. The G12V KRAS-mutated cell line, SW480, was co-treated with PBR extract and cetuximab, and proliferation and clonogenic features were evaluated. The cause of cell death was investigated using annexin V-fluorescein isothiocyanate (FITC)/propidium iodide (PI) staining, and effects on the RAS/MAPK pathway were investigated using Western blotting. In addition, PBR extract and cetuximab were administered to mice xenografted with colon cancer cells to investigate their suppressive effect on colon cancer growth.

## 2. Results

### 2.1. Phellinus Linteus on Germinated Brown Rice (PBR) Inhibits Kirsten Rat Sarcoma Viral Oncogene Homolog (KRAS)-Mutated Colon Cancer Cell Proliferation

#### 2.1.1. PBR Extract Increases the Sensitivity of a KRAS-Mutated Colon Cancer Cell Line to Cetuximab

**Figure 1:**
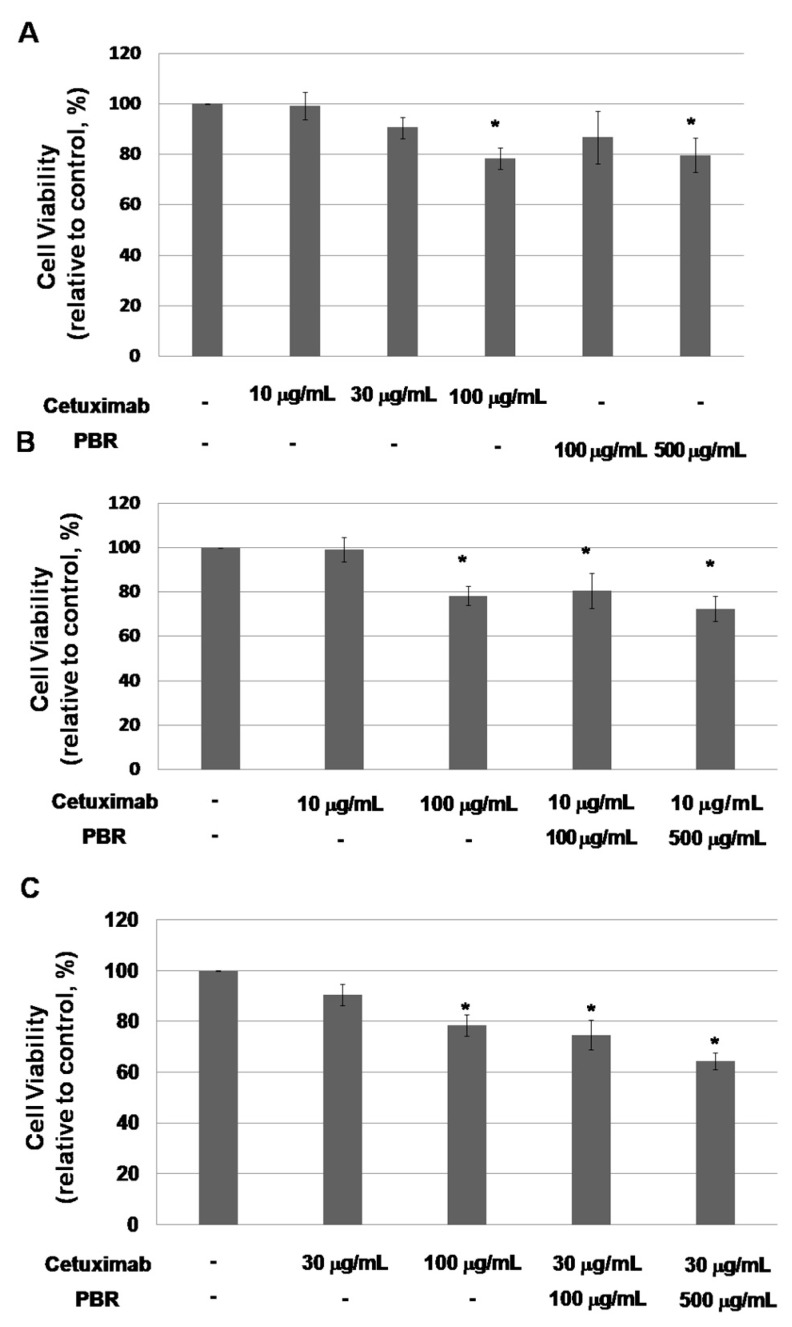
(**A**) Cell viability of SW480 colon cancer cells at 72 h with cetuximab (0, 10, 30 and 100 µg/mL) and PBR extract (100, 500 µg/mL) treatment; (**B**) SW480 colon cancer cell viability with cetuximab (10 µg/mL) and cetuximab (10 µg/mL) combined with PBR extract (100, 500 µg/mL) treatment. Cell viability was compared to that of cetuximab treatment alone, and a significant difference is denoted as * (*p* < 0.05); (**C**) SW480 colon cancer cell viability with cetuximab (30 µg/mL) and cetuximab (30 µg/mL) combined with PBR extract (100, 500 µg/mL) treatment. Cell viability was compared to that of cetuximab treatment alone (30 µg/mL) and a significant difference is denoted as * (*p* < 0.05). PBR, *Phellinus* *linteus* grown on germinated brown rice. Experiments were performed in triplicate and data are expressed as the mean ± standard error of the mean (SEM).

Among the human colorectal cancer (CRC) cell lines, a cell line with a KRAS mutation on codon 12, the KRAS^G12V^-mutated colon cancer cell line SW480, was chosen. We evaluated whether the addition of PBR extract potentiated the antitumor activity of cetuximab in this cell line. We tested a range of concentrations of cetuximab that would eventually be used in combination with PBR extract to effectively investigate their antitumor activity (Figure 1). On day 3 of culture, cell viability was barely affected by cetuximab at typically used concentrations (i.e., 10 and 30 μg/mL with 99.5 ± 5.5% and 91.0 ± 4.2% cell viability, respectively) (Figure 1A).Cell viability was lowered by an unusually high concentration of 100 μg/mL cetuximab. Under PBR treatment, cell proliferation was reduced at certain concentrations (500 μg/mL). To determine the synergic effect of PBR extract, cells were treated with cetuximab (10 and 30 µg/mL), alone and in combination with PBR extract (100 and 500 µg/mL), and cell viability was assessed after three days (Figure 1B,C). Compared to that of cetuximab alone, the combination of cetuximab and PBR extract showed significantly (*p* < 0.001) reduced cell viability (e.g., 10 μg/mL cetuximab vs. 10 μg/mL cetuximab + 100 μg/mL PBR, mean viability = 99.0 ± 5.5% vs. 80.7 ± 7.9%, *p* = 0.002). In addition, PBR suppressed the cell proliferation of KRAS wild-type colon cancer HT-29 cells (Figure S1). HT29 cell viability was affected by PBR treatment (100 and 500 μg/mL with 78.7 ± 7.4% and 72.4 ± 1.5% cell viability, respectively). When they were exposed to PBR and cetuximab, cell proliferation was significantly decreased (e.g., 30 μg/mL cetuximab vs. 30 μg/mL cetuximab + 500 μg/mL PBR, mean viability = 89.2 ± 2.4% vs. 57.2 ± 2.6%, *p* = 0.002).

#### 2.1.2. Cetuximab and PBR Extract Co-Treatment Alters Cell Morphology and Clonogenic Characteristics

**Figure 2:**
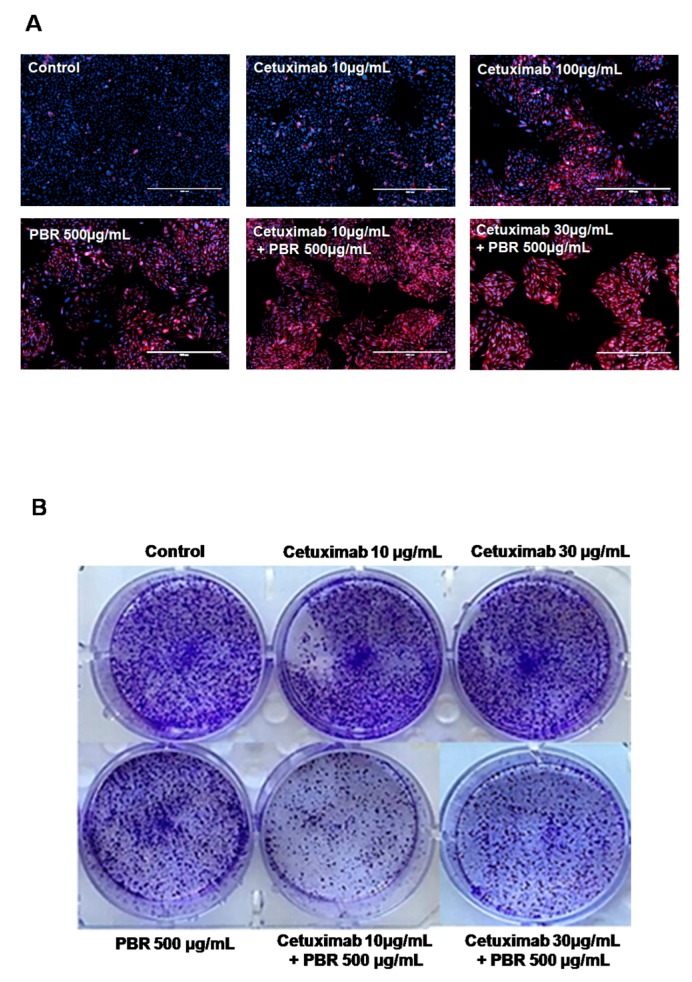
(**A**) Representative SW480 cell images after cetuximab (10, 100 µg/mL), PBR extract (500 µg/mL), or combined cetuximab and PBR extract treatment. Blue represents DAPI-stained cell nuclei, and propidium iodide-stained dead cells are red. (Scale bar = 500 µm); (**B**) Clonogenic assay images after cetuximab (10, 30 µg/mL), PBR extract (500 µg/mL), or combined cetuximab and PBR extract treatment on day 10. PBR, *Phellinuslinteus* grown on germinated brown rice; DAPI, 4′,6-diamidino-2-phenylindole.

Morphological analysis usingdead cell staining was performed. Dead cells were stained with Propidium iodide (PI) and appeared as a red color, and cell nucleiwere stained blue with DAPI during 48 h cultivation with the samples (Figure 2A). In the control group, most colon cancer cells were only DAPI-stained, which showed that most cells were viable. When cells were treated with PBR extract or cetuximab, a few PI-stained dead cells were observed. In contrast, the combined treatment of cetuximab and PBR exhibited relatively fewer cells, and most of them were red-stained dead cells (Figure 2A).

To determine the synergistic effect of the long-term anti-proliferative activity of PBR extract and cetuximab, a clonogenic assay was performed. As seen in Figure 2B, the colon cancer cell colonies were numerous in the control, and in both cetuximab (10 and 30 μg/mL) treatment groups. In contrast, reduced numbers of colonies were observed with the combined treatment of cetuximab and PBR extract (10 µg/mL cetuximab + 500 µg/mL PBR and 30 µg/mL cetuximab+ 500 µg/mL PBR).

#### 2.1.3. PBR ExtractInduced KRAS-Mutated Colon Cancer Cell Apoptosis

**Figure 3:**
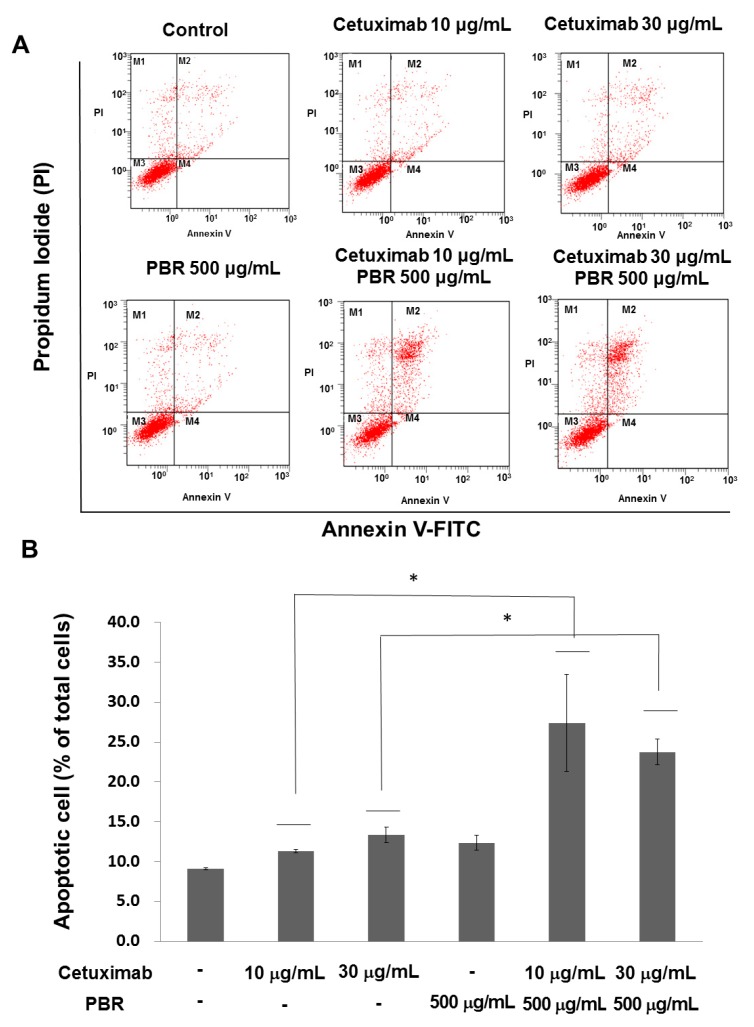
(**A**) Representative flow cytometry plots using Annexin V-FITC/PI staining for apoptosis. SW480 cells were treated for 48 h and were then stained with Annexin V-FITC/PI for flow cytometric analysis; (**B**) The percentage of apoptotic cells between cetuximab and the combination of cetuximab and PBR extract was statistically compared and significant differences are denoted by * (*p* < 0.05). PBR, *Phellinuslinteus* grown on germinated brown rice; FITC, fluorescein isothiocyanate; PI, propidium iodide. Experiments are performed in triplicate and data are expressed as the mean ± standard error of the mean (SEM).

To evaluate the effects of the combination of PBR extract and cetuximab on cell death, annexin V-FITC/PI staining and flow cytometry were performed (Figure 3). Fluorescence-activated cell sorting (FACS) analysis indicated that the combination of PBR extract and cetuximab induced apoptotic cells. For example, cetuximab treatment (10 μg/mL) induced a total of early (annexin V FITC^+^/PI^−^) and late (annexin V FITC^+^/PI^+^) apoptosis of 11.3 ± 0.23%. When combined with PBR extract, the apoptotic rate was increased to 27.4 ± 6.07%, which is similar to that of 100 µg/mL cetuximabtreatment (25.9 ± 0.94%). A similar tendency was observed at the higher cetuximab concentration (e.g., 30 μg/mL cetuximabvs. 30 μg/mL cetuximab + 100 μg/mL PBR, 13.3 ± 0.98% vs. 23.8 ± 1.64%).

### 2.2. Effect of the Combination Treatment of PBR Extract and Cetuximab on KRAS and Mitogen-Activated Protein Kinase (MAPK) Signaling Pathways

**Figure 4:**
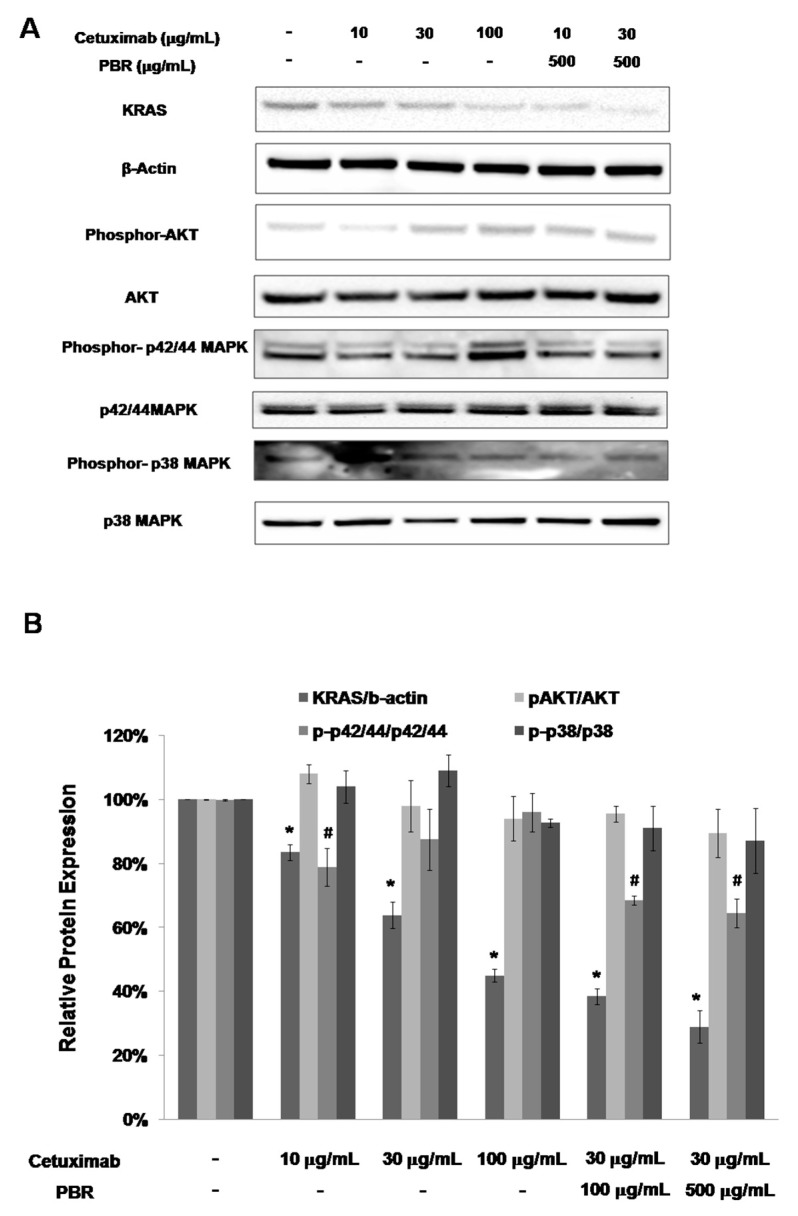
(**A**) Representative Western blot images of KRAS, β-actin, phospho-AKT/AKT, phospho-p42/44 MAPK (phospho-ERK1/2), p42/44 MAPK (ERK1/2), and phospho-p38/p38 protein expression in SW480 cells treated with cetuximab (10, 30, 100 μg/mL), PBR extract (500 μg/mL), or the combination of cetuximab and PBR extract; (**B**) Protein intensity was analyzed in triplicate. The ratios of KRAS/β-actin, phosphor-AKT/AKT, phosphor-p42/44/p42/44 and phosphor-p38/p38 were calculated. The ratio was compared to the no-treatment group and significant differences are denoted by * (KRAS/actin) and ^#^ (phosphor-p42/44/p42/44) (*p* < 0.05). Experiments are performed in triplicate and data are expressed as the mean ± standard error of the mean (SEM).

RAS protein kinases initiate the mitogen-activated protein (MAP) kinase cascade, which leads to RAF-MAPK (mitogen-activated protein kinase) pathway activations [18]. In this RAF-MAPK pathway, extracellular signal-regulated kinase (ERK)-mediated transcriptional upregulation is related to cell cycle progression and transcription, so it is directly involved in tumor progression. In fact, the phosphorylated MAPKs, ERK1 and ERK2, are observed in ~30% of human tumors. Therefore, we investigated the level of KRAS as well as MAPK, ERK1, and ERK2 protein phosphorylation after sample treatment. In addition, the phosphorylation of AKT, a kinase in a RAS cross-related pathway, was also observed. As seen in Figure 4, KRAS expression was inhibited by 10 and 30 µg/mL cetuximab compared to the control. When cells received the combined treatment of cetuximab and PBR extract, KRAS and p-p42/44 MAPK levels were reduced. As an alternative downstream target of KRAS, the phosphorylation of AKT and p38 was not changed by sample treatment. Based on these results, the combined treatment of PBR extract and cetuximab appears to reduce MAPK signaling by reducing KRAS expression.

### 2.3. PBR Extract Inhibits Xenografted Colon Cancer Growth In Vivo

**Figure 5:**
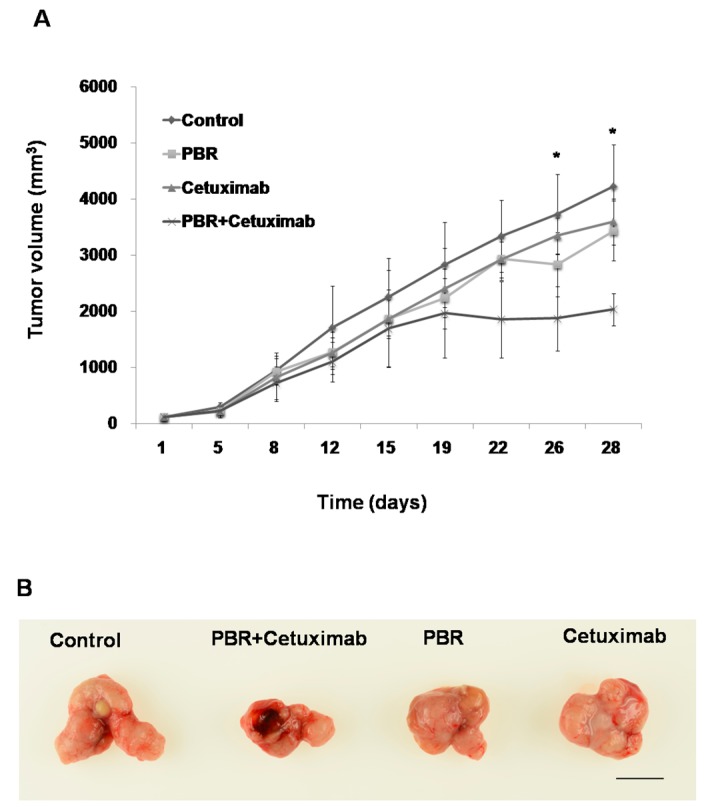
(**A**) Tumor volume changes in SW480 xenografted mice treated with control (saline), cetuximab (10 mg/kg in PBS), PBR extract (400 mg/kg/day), or the combination of cetuximab and PBR extract. Tumor volumes were compared to those of the control treatment, and a significant difference is denoted as * (*p* < 0.05); (**B**) Representative SW480 xenograft tumors resected on day 26 showing the difference in tumor volumes between individual PBR extract or cetuximab treatment, and cetuximab and PBR extract combined treatment. Scale bar = 1 cm. Data are expressed as the mean ± standard error of the mean (SEM).

To investigate whether the enhanced antitumor activity of combined PBR extract and cetuximab treatment is also observed in vivo, we implanted SW480 tumors in mice. The combination of cetuximab and PBR extract produced a statistically significant reduction in tumor volume, compared with that of cetuximab alone, in the KRAS-mutant SW480 xenografts (mean tumor volume on day 26 = 3344 ± 572 mm^3^ vs. 1870 ± 571 mm^3^, cetuximab vs. cetuximab + PBR, *p* = 0.011) (Figure 5). In contrast, there was no significant antitumor activity with the PBR extract or cetuximab alone. Body weights of mice were recorded every threedays during the experiments. PBR extract feeding appeared tolerable since these mice had similar body weights compared to those of the control groups. Therefore, the addition of PBR extract to cetuximab suppressed KRAS-mutated colorectal tumor growth invivo.

## 3. Discussion

Approximately 30–40% of patients with colon cancer have a KRAS mutation, and they cannot benefit from cetuximab therapy [5]. In this study, we demonstrated that PBR extract treatment could sensitize KRAS mutant cells to cetuximab therapy in vitro and in vivo. In Figure 1, G12V KRAS mutant SW480 cell line growth was evaluated with cetuximab, PBR extract, or acombined treatment of the two. When cetuximab and PBR extract were combined, enhanced antitumor activity (as assessed by cell viability and clonogenic properties) in G12V KRAS mutant colon cancer cells was observed, which was caused by apoptosis. From the RAS/MAPK pathway study, it was concluded that PBR extract can overcome cetuximab resistance via the modulation of KRAS expression and the induction of apoptosis by altering the phosphorylation of MAPK. In a mouse model, the growth of KRAS-mutant xenografted tumors was suppressed when PBR extract was added to cetuximab during treatment.

Several studies have tried to overcome the cetuximab resistance of KRAS-mutated colon cancers, including those using microRNA [19], L-ascorbic acid, lauric acid, cisplatin, dasatinib, and simvastatin. Cetuximab sensitization was achieved with dasatinib and simvastatin [20,21]; however, the requirement of adding more drugs limited the clinical application. Recently, the induction of sensitivity with natural products, such as ascorbic acid and lauric acid, was studied. Weng et al. demonstrated that lauric acid significantly improved sensitivity to cetuximabwith KRAS and BRAF mutants [22]. Lauric acid can be easily obtained from natural food, and it is reportedly harmless to the cardiovascular system. Ascorbic acid can abrogate cetuximab resistance in mutant KRAS human colon cancers [23]. This enhanced antitumor activity of cetuximaboccurred via the inhibition of cellular proliferation or the induction of apoptosis in various cancer cell types.

In the present study, adding PBR extract increased cell sensitivity to cetuximab, achieving an equivalent suppressive effect with lower cetuximab concentrations. That might result from functional compounds in the PBR extract. Compared to the levels in whole *P. linteus*, γ-aminobutyricacid (GABA) and β-glucan concentrations are high and ergosterol peroxide is present in the PBR extract. The GABA and β-glucan concentrations were found to be 141.14 and 29.20 mg/g on a dry weight basis, respectively, which are four-fold and 7.5-fold higher, respectively, than those found in whole *P. linteus* (CARI homepage, http://www.cellacti.com/sub03_01.php). The ergosterol peroxide content in PBR extract was found to be 1200 ppm, but it wasnot found in whole *P. linteus* [24]. GABA is the main inhibitory neurotransmitter in the vertebrate brain and is involved in the proliferation, migration, and survival of neurons in fetal and newborn mammalian brains [25]. Interestingly, GABA inhibits the migration of colon cancer cells, which delays cancer invasion and metastasis. GABA has an inhibitory action on cancer cell proliferation and stimulates cancer cell apoptosis [26]. Ergosterol peroxide is an antitumor sterol produced by mushrooms that shows inhibitory activity against human colon cancer cells [27]. Further, β-glucan provides beneficial health effects that are derived from its antitumor, anti-infectious disease, and anti-atherosclerosis properties [28]. Interestingly, β-glucan was able to enhance antitumor effects by synergizing with monoclonal antibodies against various antigens, tumor types, or tumor sites [29]. Since β-glucan showed synergistic effects with EGFR [30], the increased sensitivity of cetuximab combined with PBR extract could result from the high concentrations of β-glucan in the PBR extract. Investigating the role of β-glucan as a sensitizer of cetuximab resistance deserves further study.

To our knowledge, this is the first report to show the potential role of PBR extract to overcome cetuximab resistance in KRAS-mutant colon cancer. Our current study combined the antitumor monoclonal antibody drug, cetuximab, and the functional food, PBR extract, to act synergistically against KRAS-mutant colon cancer cells. This synergistic effect could result from β-glucan in the PBR extract. From the data provided, it appears that PBR extract can sensitize KRAS mutant tumors to cetuximab toxicity. PBR is a food that is consumed in daily life. Therefore, these findings highlight a straightforward and practical therapeutic for CRC patients with KRAS mutations, which is currently an unmet need.

## 4. Materials and Methods

### 4.1. Preparation of PBR Extract

PBR was provided by Cell Activation Research Institute (CARI; Kyungji-Do, Korea). Dried, 6-week-old PBR was ground into a fine powder using a grinder. The powder (2 kg) was extracted with ethanol and ethyl acetate at 20–25 °C. After filtration, the ethanolic extracts were dried with a rotary evaporator under vacuum, and the dried extract was stored at −20 °C. Authenticated voucher specimens of PBR (Kucari 0905) were deposited in the CARI (Kyungji-Do, Korea).

### 4.2. Cell Viability by MTS Proliferation Assay and DAPI/PI Staining

The SW480and HT-29cell line was purchased from American Type Culture Collection (ATCC, Rockville, MD, USA) and were cultured in regular media consisting of RPMI-1640 (Invitrogen, Carlsbad, CA, USA), 10% fetal bovine serum (FBS; Invitrogen), and 100 U/mL penicillin-streptomycin (Sigma, St. Louis, MO, USA). For the cell viability assay, cells were seeded into 96-well plates (20,000 cells/well) in triplicate. Twenty-four hours later cells were treated with samples (10 and 30 µg/mL cetuximab, 100 µg/mL PBR extract, or their combination) and incubated for 3 days. At the end of the incubation, cell viability was determined by adding (3-(4,5-dimethylthiazol-2-yl)-5-(3-carboxymethoxyphenyl)-2-(4-sulfophenyl)-2*H*-tetrazolium, inner salt (MTS) reagent for 1 h at 37 °C, according to manufacturer’s instructions (MTS Assay, Promega, Fitchburg, WI, USA). Absorbance readings were acquired at 490 nm (Model 550; Bio-Rad, Hercules, CA, USA), and the reduction in cell growth was calculated as a percentage of absorbance in the absence of treatment.

### 4.3. Live/Dead Staining

For microscopic observation, cells were fixed with 4% paraformaldehyde (Sigma) in phosphate-buffered saline (PBS for 10 min at room temperature, and were then washed with PBS three times. Fixed cells were stained with 1 µg/mL of DAPI (Sigma) for 10 min at room temperature. The stained cells were then observed under an inverted fluorescence microscope (EVOS^®^, Thermo Fisher Scientific, Waltham, MA, USA).

### 4.4. Clonogenic Assay

Cells were seeded in 6-well plates (2000 cells/well) for the colony formation assay. Following 24h incubation, cells were treated with cetuximab, PBR extract, or their combination. After 10 days, cells were washed with PBS and fixed with 4% paraformaldehyde for 5 min. The colonies were stained with 1% crystal violet (Sigma) for 30 s, and were rinsed three times with PBS followed by air-drying.

### 4.5. Flow Cytometry

The extent of SW480 apoptosis after treatment was evaluated using an Annexin V-FITC/PI apoptosis detection kit (BD Biosciences, San Jose, CA, USA), according to the manufacturer’s instructions. SW480 colon cancer cells were seeded in a six-well plate (5 × 10^5^ cells/well) overnight, and were then treated with cetuximab (10, 30 or 100 μg/mL), PBR extract (100 μg/mL), or a combination of cetuximab and PBR extract. After 48 h, cells were harvested, washed in ice-cold PBS, and collected by centrifugation at 500× *g* for 10 min. Cells were stained simultaneously with FITC-labeled annexin V (5 µL) and PI (5 µL) at room temperature for 10 min, protected from light. Stained cells were analyzed using a fluorescence-activated cell sorter flow cytometer (FC 500 Series Flow Cytometry, Beckman Coulter, Indianapolis, IN, USA). At least 10,000 cells were used for each analysis, and experiments were performed in triplicate.

### 4.6. Western Blotting

Cells were washed three times in cold PBS, and protein lysates were obtained by extraction with RIPA buffer containing protease inhibitors (Sigma). Proteins were separated on 10% pre-cast SDS-PAGE gels (Bolt™ 4–12% Bis-Tris Plus Gels) (Invitrogen), and were then transferred using the iBlot system (Life Technology). Membranes were incubated with the following primary antibodies: rabbit anti-KRAS, anti-phospho-p42/44 MAPK (ERK1/2), anti-p42/44 (ERK1/2), anti-phospho-Akt, anti-phospho-p38 MAPK, and anti-p38 (all from Cell Signaling, Danvers, MA; used at 1:1000 dilution); rabbit anti-Akt (1:1000, Santa Cruz Biotechnology, Dallas, TX, USA); and mouse anti-β-actin (1:4000, Sigma). Membranes were then incubated with horseradish peroxidase-conjugated goat anti-rabbit or anti-mouse antibodies (Pierce Biotechnology Inc., Rockford, IL, USA), and detected using a SuperSignal^®^ West Femto enhancer kit (Pierce). Protein intensity was analyzed by Image J (NIH, Bethesda, MD, USA) in triplicate. The ratios of KRAS/β-actin, phosphor-AKT/AKT, phosphor-p42/44/p42/44 andphosphor-p38/p38 were calculated. The expression level was compared to no treatment group.

### 4.7. Animal Experiment

The inhibitory effect of cetuximab, PBR extract, and their combination on colon cancer growth was investigated in an animal model. Athymic nude mice (sixweeks, male) were purchased from BioToxTech animal center (Cheongju, Korea). All animals were handled following the guidelines of the Institutional Animal Care and Use Committee (IACUC) at Silla University. Animal protocol was reviewed and approved by IACUC at 01 December 2016 (#160782). Mice were injected bilaterally in the dorsal flank with SW480 cells (5 × 10^6^ cells/site) [31]. The mice were treated twice per week with an intraperitoneal injection of 10 mg/kg cetuximab in PBS. PBR extract was administered by oral gavage (400 mg/kg/day) daily. Once tumors reached 100 mm^3^, mice were started on their respective treatments (cetuximab, PBR extract, combination of cetuximab and PBR extract, or saline control, *n* = 7/group). Tumor volumes were calculated using the following formula: *V* = (*L* × *W*^2^)/2, where *V* = volume, *L* = length and *W* = width. The mice were euthanized on day 23, and tumor viscera were imaged with a digital camera.

### 4.8. Statistical Analysis

Data are expressed as the means ± standard error of the mean (SEM). Statistical analyses were performed by one-way analysis of variance (ANOVA), using the SPSS software, version 12 (SPSS Inc., Chicago, IL, USA). Differences were considered significant at *p* < 0.05.

## Supplementary Materials

Supplementary materials can be found at www.mdpi.com/1422-0067/18/8/1746/s1.
