# Supplementary figures and images for: Phellinus linteus Grown on Germinated Brown Rice Increases Cetuximab Sensitivity of KRAS-Mutated Colon Cancer
Source: Int J Mol Sci. 2017 Aug 11;18(8):1746. doi: 10.3390/ijms18081746 (PMC5578136; doi:10.3390/ijms18081746)

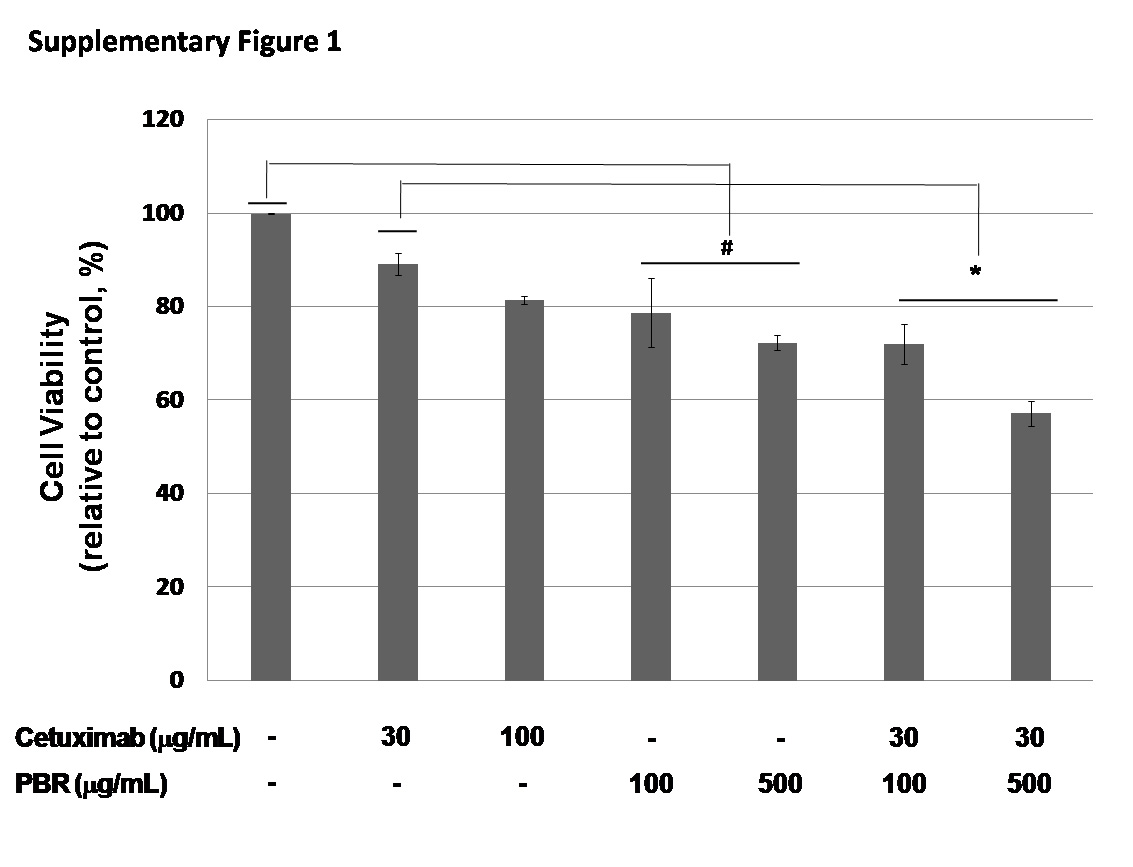

Supplement: Supplementary file 1 [file ijms-18-01746-s001.jpg]
